# Can singular examples change implicit attitudes in the real-world?

**DOI:** 10.3389/fpsyg.2013.00594

**Published:** 2013-09-05

**Authors:** Leslie E. Roos, Sophie Lebrecht, James W. Tanaka, Michael J. Tarr

**Affiliations:** ^1^Department of Cognitive, Linguistic and Psychological Sciences, Brown UniversityProvidence, RI, USA; ^2^Department of Psychology, University of OregonEugene, OR, USA; ^3^Center for the Neural Basis of Cognition, Carnegie Mellon UniversityPittsburgh, PA, USA; ^4^Department of Psychology, University of VictoriaVictoria, BC, Canada; ^5^Department of Psychology, Carnegie Mellon UniversityPittsburgh, PA, USA

**Keywords:** face processing, affective valence, affective priming, implicit attitudes, other-race effect

## Abstract

Implicit attitudes about social groups persist independently of explicit beliefs and can influence not only social behavior, but also medical and legal practices. Although examples presented in the laboratory can alter such implicit attitudes, it is unclear whether the same influence is exerted by real-world exemplars. Following the 2008 US election, Plant et al. reported that the Implicit Association Test or “IAT” revealed a decrease in negative implicit attitudes toward African-Americans. However, a large-scale study also employing the IAT found little evidence for a change in implicit attitudes pre- and post-election. Here we present evidence that the 2008 US election may have facilitated at least a temporary change in implicit racial attitudes in the US. Our results rely on the Affective Lexical Priming Score or “ALPS” and pre- and post-election measurements for *both* US and non-US participants. US students who, pre-election, exhibited *negative* associations with black faces, post-election showed *positive* associations with black faces. Canadian students pre- and post-election did not show a similar shift. To account for these findings, we posit that the socio-cognitive processes underlying ALPS are different from those underlying the IAT. Acknowledging that we cannot form a causal link between an intervening real-world event and laboratory-measured implicit attitudes, we speculate that our findings may be driven by the fact that the 2008 election campaign included extremely positive media coverage of President Obama and prominently featured his face in association with positive words—similar to the structure of ALPS. Even so, our real-world finding adds to the literature demonstrating the malleability of implicit attitudes and has implications for how we understand the socio-cognitive mechanisms underlying stereotypes.

## Introduction

Implicit biases are attitudes or preferences that are automatic and occur without conscious control (Greenwald et al., 1998). Over the past decade, tests of implicit racial attitudes have found that participants (on average) possess negative associations toward black faces and African-Americans (Phelps et al., 2000; Phelps, 2001; Richeson et al., 2003; Lieberman et al., 2005; Trawalter et al., 2008). Critically, these findings hold across a variety of implicit measures (Fazio et al., 1995; Greenwald et al., 1998; Lebrecht et al., 2009), as well as the race of the participant (Nosek et al., 2002). At the same time, these attitudes are apparently malleable and can be altered at the individual level through laboratory manipulations that rely on training or priming (Kawakami et al., 2000; Dasgupta and Greenwald, 2001; Correll et al., 2007; Lebrecht et al., 2009). However in studies without direct efforts or modifications to reduce bias, participants consistently maintain a negative association to black faces (Nosek et al., 2002; Lieberman et al., 2005; Green et al., 2007; Schmidt and Nosek, 2010).

With respect to short-term changes in attitudes, recent socio-cognitive studies have elucidated many of the factors that can alter an individual participant's implicit attitudes. A comprehensive review by Blair and colleagues includes a meta-analysis of such studies and concludes there is a strong case for the short-term malleability of attitudes in response to the perceiver's motives and strategies (Blair, 2002). Certain factors that can moderate bias, such as self or social motives or threatened self-esteem, provide relevant theory regarding suppression of automatic stereotypes. In particular, priming studies using salient stereotypic vs. non-stereotypic social examples (e.g., African-Americans in gang setting vs. African-Americans at an outside BBQ) or salient positive vs. negative individual exemplars (e.g., Bill Cosby vs. OJ Simpson) have both been shown to modulate expression of African-American stereotypes, (Dasgupta and Greenwald, 2001; Wittenbrink et al., 2001). Such findings point to the potential of exposure for modulating implicit attitudes over the short-term. Moreover, the fact that examples of prominent individuals have some efficacy hints at the long-term factors that may be able to influence implicit attitudes within real-world populations given the right confluence of conditions (e.g., saliency, degree of counter-stereotypicality, widespread awareness of the exemplar, etc.).

In this paper we build on such results in describing a recent change in implicit attitudes that apparently arose naturally within the general population. Because of the real-world nature of our findings, it is impossible for us to definitively identify any causal relationship between changes observed in the laboratory over time and real-world events. Indeed, there has been some debate in the literature as to whether such effects exist at all (Schmidt and Nosek, 2010). At the same time, our data is strengthened by the fact that it was coincidentally collected *prior to* and *following* a highly-salient and widely-publicized social milestone in US history—the 2008 election of the first African-American US President. As such, we base our speculative interpretation of our results on recent socio-cognitive studies (Cooper, 2009) and econometric analyses (Dubner, 2011) that have attempted to address the impact of this event. To help account for why a singular counter-stereotypic exemplar might have a significant impact on implicit racial attitudes, we propose a cognitive-process account for how implicit attitudes are both initially anchored and how they may change over time. As one component, we posit that racial attitudes implicitly develop in conjunction with the automatic evaluation of human faces along many non-perceptual dimensions. That is, when observers visually perceive a face they unconsciously assign it a positive or negative “valence” based on their prior associations with that face and faces of a similar category (e.g., faces of the same race, age, sex, etc.). This evaluation of visual input and valence association occurs very early and ubiquitously in visual processing (Bar et al., 2006; Barrett and Bar, 2009). Consequently, the resultant associations, including valence, have ample opportunity to influence processing within other social and cognitive systems.

Faces, as well as objects more generally, accumulate affective information both from environmental factors (e.g., context and the opinions of others; Bar et al., 2006) and from one's affective reactions (e.g., one's somatic responses; Barrett and Bar, 2009). Furthermore, these affective associations directly generalize to the interpretation of new objects or new faces that are perceptually similar to familiar valenced objects or faces. This model is supported by the recent finding that participants are able to: (a) learn that particular valences go with particular faces; (b) (more interestingly) generalize these valences to novel faces that are morphed to look like the learned-valence faces. In particular, novel faces morphed with positive exemplars were judged more positively than novel faces morphed with neutral or negative exemplars (Verosky and Todorov, 2010).

Such effects may be particularly strong with respect to other-race attitudes given the well-established visual other-race effect, in which other-race faces are judged to be more perceptually similar to one another as compared to own-race faces (Malpass and Kravitz, 1969; Ferguson et al., 2001; Tanaka and Pierce, 2009). Given that implicit attitudes can arise from the automatic evaluation of a face, and that observers transfer the resultant valence to novel faces that are visually similar (Verosky and Todorov, 2010), we posit that implicit racial attitudes persist because once a particular association or stereotype arises for one face, it is generalized to other, perceptually-similar faces. Supporting this conjecture, we recently found that reducing the visual other-race effect through perceptual expertise training—so that post-training other-race faces look perceptually more different from one another—concomitantly reduced implicit racial biases within individual participants (Lebrecht et al., 2009).

**Figure 1:**
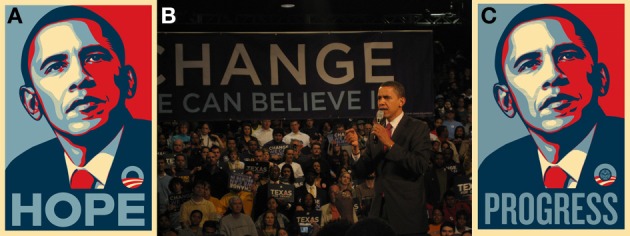
**Sample media from Barack Obama's 2008 US presidential campaign**. The visual pairing of Barack Obama's face with highly positive words was ubiquitous during the campaign and may have contributed to the observed change in US participants' valence associations with black faces. There is an interesting similarity between the face-word pairings used in the campaign posters and the positive word trials in the ALPS measure of implicit bias. **(A)** Campaign poster presenting the face of Barack Obama above “HOPE” (which has a positive word rating score of 7.05, where 0 = most negative, and 9 = most positive—as measured by the ANEW corpus); **(B)** Barack Obama next to “CHANGE” (ANEW does not have a rating for “Change”); **(C)** Barack Obama's face above “PROGRESS” (which has a positive word rating score of 7.73). **(A,C)** Are Barack Obama 2008 campaign posters created by Rhode Island artist Shepard Fairey <http://obeygiant.com/>. **(B**) Is in the public domain and was provided via http://commons.wikimedia.org/wiki/File:Barack_obama_houston.JPG.

With respect to the shift in implicit attitudes we report here, it is our speculation that the extensive media coverage and the direct advertising^1^ surrounding Barack Obama's presidential campaign and election generated a positive counter-stereotype that generalized to other black faces. Throughout the 2008 US presidential campaign images of President Obama's *face* were consistently presented alongside written messages of optimism such as “HOPE” and “CHANGE” (using salient and unique facial imagery; Figure 1). For the most part, the media coverage associated with President Obama has continued to be counter-stereotypic from that often associated with African-Americans. As but one example, a study of US news broadcasts in the 1990s found that 46% of news stories involving African-Americans presented them as a “threat to or non-contributing victims of American society” (Entman, 1994). Similarly, Eberhardt et al. (2004) found that media portrayals are strongly reflected in individual participant's associations with African-Americans. For instance, exposing participants to black faces lowered the perceptual threshold for detecting images of crime-relevant objects (e.g., guns, knives) and exposing participants to crime-relevant objects primed attention to black faces. Thus, it appears plausible that the departure from the norm in media coverage associated with Obama's election campaign may have altered the generic valences US residents associate with black faces and, in turn, altered the automatic affective evaluation of black faces as measured in laboratory assessments of implicit racial bias.

Of note, election campaign coverage was particularly prominent in the US college environment in that much of President Obama's campaign targeted a younger voting population with new media (Cave, 2008). Furthermore, Shepard Fairey's (a Rhode Island artist) iconic images were highly present on Brown's college campus in the form of posters, t-shirts, and stickers. A poll by the Brown Daily Herald on November 3rd, 2008 found that 86.1% of Brown University students supported Barack Obama and over 85% of these individuals were registered to vote (Liss, 2008). The Herald further reported that on election night the campus “exploded in jubilant spontaneous celebration” with students pouring onto the main green, then down to the State House, while students lit fireworks in victory, chanting “Yes, We Can!” on the steps of public buildings (Fedor, 2008). This same pattern was repeated throughout the US: 66% of college-age voters supported President Obama (31% supported Senator McCain) and over 3.4 million more people aged 18–29 voted in 2008 than in the previous US presidential election (Cave, 2008).

Whether such a shift in the media portrayal of a racial group (or a prominent individual from that group) may influence implicit racial attitudes in the real-world is an open question that is not easily addressed using traditional laboratory methods (Schmidt and Nosek, 2010). However, by happenstance we had access to the measured implicit racial attitudes of two different participant populations that most likely varied in their exposure to President Obama's presidential campaign and concomitant media coverage: one group of students in the US and the other group of students in Canada. As such, our study is partially a matter of chance—we had collected measures of implicit racial bias toward African-Americans in *both groups* prior to the 2008 US presidential election campaign and collected similar data post-election in both groups given that we had some indication that there was a shift in attitudes within our US participants. That is, making the somewhat controversial assumption that the popular media, encompassing both advertising and news coverage, did have the capacity to alter implicit attitudes, we wondered whether participants from the US were more likely to show a shift in attitudes following the presidential election, while participants from Canada were more likely to maintain pre-election attitudes.

For both groups of participants, we measured implicit racial attitudes using the Affective Lexical Priming Score (ALPS) (Lebrecht et al., 2009). ALPS uses response times from a lexical decision task to assess a participant's implicit racial attitudes—in many ways ALPS is similar to the Bonafide Pipeline (Fazio et al., 1995) and is, in some ways, similar to the IAT (Greenwald et al., 1998). However, both of these commonly-used measures of implicit attitudes make *direct reference* to valence in their tasks. For example, in the race IAT (Greenwald et al., 1998) participants make categorical decisions about African-Americans and Caucasian Americans while the two button presses are shared with the affective categories “good” and “bad”; in the Bonafide Pipeline (Fazio et al., 1995) participants make categorical decisions directly on words, judging them as either “positive” or “negative.”

Critically, ALPS asks participants to perform a lexical decision task that is *unrelated to valence* except indirectly via the affective connotations of the semantically-unrelated words. Lexical decision tasks, in which participants are presented with a prime and then decide whether the target letter-string is a real word or a non-sense word, have been widely used to study semantic priming (Meyer and Schvaneveldt, 1971). If the prime word is *semantically related* to the target (presumably as defined by the organization of one's lexicon), participants are faster to make the word/non-sense word decision as compared to when the prime word is semantically unrelated (Meyer and Schvaneveldt, 1971). We suggest that the same basic set of psychological processes underlies the priming of the target words in ALPS, except that the priming due to the image prime is now occurring along an affective valence, rather than semantic, dimension. That is to say, if a participant evaluates a face as negative then they are likely to be faster to make a word/non-sense word decision if the target word is likewise relatively negative (as compared to other words in a participant's lexicon). In ALPS the *valence* of the prime relates to the *valence* of the target, irrespective of semantics/meaning. For example, a face perceived as negative can prime the target word “cancer” because they are both negative in valence; faces and cancer are otherwise semantically unrelated. Also of note, in our use of ALPS positive and negative words are embedded in the context of many neutral and non-words, leaving the majority of participants naïve (by self-report) to the goals of our experiment, and arguably rendering ALPS highly implicit for most participants. As will be discussed later, we view these specific features of the socio-cognitive mechanisms underlying ALPS as essential in accounting for why we apparently saw shifts in implicit racial attitudes post-election, but a detailed, extremely large-*N* analysis of IAT results revealed no such shift (Schmidt and Nosek, 2010).

In that ALPS is a relatively novel method with few published applications it is worth asking how stable are the positive and negative valence effects found using ALPS (in contrast, the IAT has been used in literally 100's of studies with 1,000,000's of samples)? Of relevance is that we have used ALPS with a wide variety of common objects independently rated to be carrying either positive or negative valence. Moreover we have used common objects carrying either strong valences (e.g., IAPs pictures) or “micro-valences” (e.g., teacups, kettles, chairs, etc.). Critically, results from ALPS using these common objects are consistent with several different methods for obtaining valence ratings. Overall these results indicate that ALPS is effectively measuring implicit attitudes, but it is the fact that we observed consistent responses for micro-valenced objects that suggests that ALPS is stable for more subtle valence differences, such as those seen within a race (Lebrecht, 2012).

## Materials and methods

### Ethics statement

All pre- and post-election participants gave informed written consent and were self-reported Caucasians over the age of 18. All protocols were approved and conducted in accordance with the Institutional Review Boards at either Brown University or the University of Victoria.

### Participants

#### Pre-election

Thirty-four participants were recruited from Brown University and Providence from 08/02/07–11/05/07. Twenty-four participants were recruited at the University of Victoria, Canada from (11/01/07—06/30/08). Note that the date of the US presidential election was 11/04/08.

#### Post-election

Thirty participants were recruited from Brown University, Providence RI from 04/14/09–05/07/09; one participant was excluded from all reported analyses due to highly variable response times that fell more than two standard deviations away from the group mean, leaving an *N* of 21. Thirty-eight participants were recruited from the University of Victoria, Canada from 06/17/09 to 07/17/09; one participant was excluded because they self-identified as Asian following participation in the study^2^ and one participant was excluded due to a failure to follow instructions during the testing, leaving an *N* of thirty-six.

### Stimuli

#### Pre-election

Pre-election faces (Providence): Image primes consisted of 144 gray-scale male and female face images of African-American, and Caucasian individuals selected from the Tarrlab face database (www.face-place.org). These faces were normalized across race for luminance using an early version of the SHINE toolbox (Willenbockel et al., 2010). Faces displayed neutral expressions and were spatially normalized to an oval template in order to remove any external cues that could influence face perception (Figure 3).

**Figure 2:**
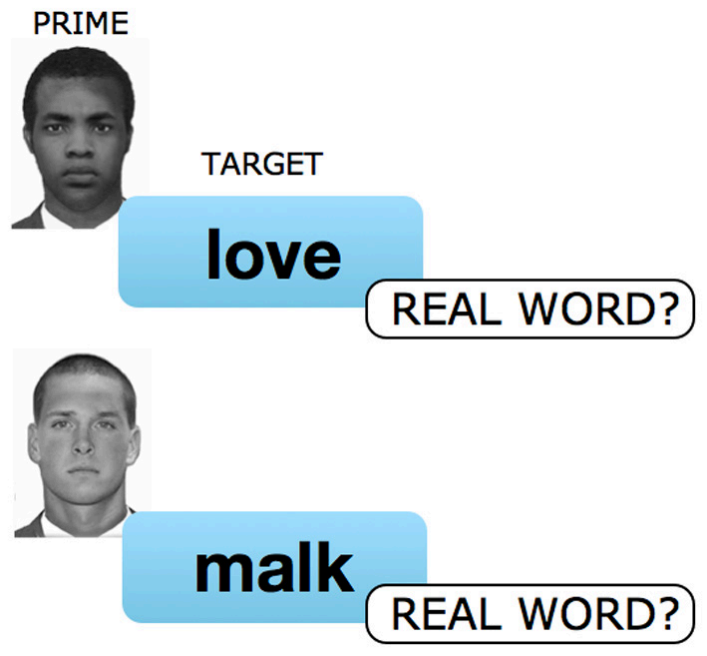
**ALPS (Affective Lexical Priming Score)**. On each trial participants were initially presented with either a black or white face image prime displayed for 250 ms. Following a 200 ms inter-stimulus interval (ISI), a letter-string was presented that was either a real word or a non-sense word. Participants were instructed to decide whether the letter-string was a real word or a non-sense word (i.e., they were naïve to the true goals of the experiment). The real words were positive (e.g., “love”), negative (e.g., “hate”), or neutral (e.g., “tree”). Each letter-string remained on the screen until the participant responded word/non-word using one of two computer keys.

**Figure 3:**
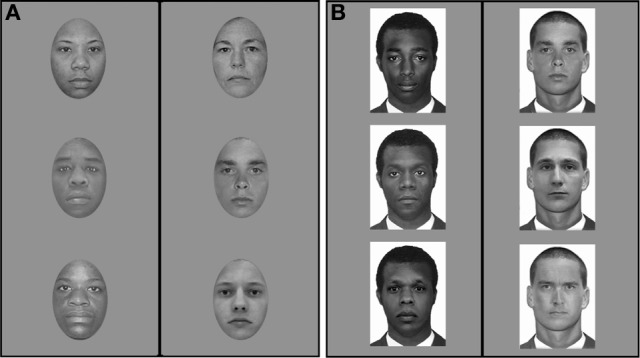
**Face images used as primes. (A)** Examples of face images used as primes in the ALPS study run in the US prior to the 2008 presidential election—sample black faces are shown in the left column, sample white faces are shown in the right column. **(B)** Examples of face images used as primes in the ALPS studies run in Canada prior to the 2008 presidential election and in the US and Canada following the 2008 presidential election—sample black faces are shown in the left column, sample white faces are shown in the right column.

Pre-election faces (Victoria): Image primes consisted of 144 gray-scale male face images of African-American, Caucasian, and Chinese individuals developed in the VizCogLab (and used in Tanaka and Pierce, 2009) taken originally from the Department of Corrections face databases from the states of Florida, Arkansas, Georgia, and Kansas. Internal face features were digitally placed in a standard face template with identical hairstyle, face contour, and clothing (Figures 2, 3). External cues (e.g., hairstyle, clothing) were kept constant. Again, these faces were normalized across race for luminance using the SHINE toolbox (Willenbockel et al., 2010).

Pre-election words (Providence and Victoria): Letter-strings consisted of 144 words divided equally between non-words and real-words (Figure 2). The real-words were divided equally between positive, negative, and neutral words taken from the IAT (Greenwald et al., 1998), the Bonafide Pipeline (Fazio et al., 1995) and an affective word website: www.winspiration.com (for a complete word list see Table 1). Word length and frequency were matched across all conditions, and valence across relevant conditions. The experimenters generated the non-words and then a third party checked to ensure they were: (a) pronounceable; (b) did not sound like any pre-existing word.

**Table 1:** **Pre-election word set**.

**Positive**	**Negative**	**Neutral**	**Non-words**	**Non-words**	**Non-words**
Excellent	Hostile	Tree	Lexolous	Tistile	Reet
Brilliant	Aggressive	Building	Britalent	Gengotten	Baulting
Delightful	Abandon	Bottle	Gengerful	Mandon	Tottle
Desirable	Awful	Walk	Sectible	Watful	Malk
Fabulous	Disturbing	Temperature	Baggerment	Listbing	Whelkature
Likable	Horrible	Think	Kirkable	Raustible	Kinget
Nice	Inferior	Examine	Keecy	Jesterior	Lexou
Pleasant	Irritating	Breakfast	Lepselant	Merritating	Trakefas
Superior	Nasty	Shelves	Periors	Prasty	Slewe
Wonderful	Offensive	Computer	Nounterful	Neffentive	Lequoter
Joy	Repulsive	Journal	Jop	Lepenting	Mourmal
Love	Rotten	Plant	Vove	Bentotten	Patal
Peace	Terrifying	Radiator	Cept	Tettifying	Raliador
Pleasure	Upsetting	Task	Lepeasure	Pusetting	Rask
Glorious	Agony	Left	Gloringous	Bagony	Wheft
Laughter	Terrible	Movement	Hawning	Tectible	Booment
Happy	Distress	Television	Papney	Listress	Relifishion
Kind	Failure	Wall	Denk	Matilure	Nall
Great	Evil	Water	Treget	Mevil	Preat
Exquisite	Hurt	Door	Exquenite	Thurt	Linlow
Charming	Hate	Poster	Marming	Vate	Loster
Adore	Disgusting	Discuss	Radore	Gelusting	Rolluss
Appealing	Rage	Observe	Popealing	Lage	Finserve
Attractive	Angry	Notice	Ractive	Ditase	Foutice

Complete list of positive words, negative words, neutral words, and non-words used as prime targets in the lexical decision component of ALPS run prior to the 2008 US presidential election.

#### Post-election

Post-election faces (Providence and Victoria): Image primes consisted of 630 male face images randomly drawn, within race condition, from 750 gray-scale male face images of African-American, Chinese, and Caucasian individuals developed in the VizCogLab (and used in Tanaka and Pierce, 2009) taken originally from the Department of Corrections face databases from the states of Florida, Arkansas, Georgia, and Kansas. Internal face features were digitally placed in a standard face template with identical hairstyle, face contour, and clothing (Figures 2, 3). External cues (e.g., hairstyle, clothing) were kept constant. Luminance was normalized within each racial group. Note that although these face images were different from those face images used pre-election in Providence, both stimulus sets were highly controlled with respect to the presence of external cues, expression, and luminance (to compare, see Figure 3). At the same time, it is important to acknowledge that the use of different face images may have influenced our results in unintended ways. For example, it is possible that the African-American faces used pre-election in Providence were viewed more negatively that the African-American used post-election. However, this would not explain the differences we observed post-election between US and Canadian participants. We return to this point in the General Discussion.

Post-election words (Providence and Victoria): Letter-strings consisted of 630 words divided equally between non-words and real words. Real-words were likewise divided equally between positive, negative, and neutral words from the ANEW corpus (Bradley and Lang, 1999). Word length and frequency were matched across all conditions, and valence across relevant conditions (for a complete word list see Table 2). Non-words were generated using the ARC non-word database (http://www.maccs.mq.edu.au/~nwdb/), and checked by a third party to ensure they were: (a) pronounceable; (b) did not sound like any pre-existing word. As with the use of different face sets across conditions, it is possible that this shift between the word sets may have affected our results. However, while we can create a scenario in which different sets of face images for a given race are regarded more positively or negatively, it is difficult to envision an account whereby the second word set used for *both* participant groups post-election would *differentially* influence how individuals in those two groups performed lexical decisions.

**Table 2:** **Post-election word set**.

**Positive**	**Negative**	**Neutral**	**Non-words**	**Non-words**	**Non-words**
Vacation	Dead	Banner	Trurfs	Brids	Sluild
Wealthy	Ugly	Cabinet	Ghlapsed	Karls	Smach
Miracle	Fearful	Item	Rask	Nazed	Stibe
Excellence	Hell	Context	Skrarge	Scarque	Trorced
Fireworks	Fat	Tank	Thwof	Thett	Sponde
Passion	Abuse	Industry	Plooched	Thute	Calp
Puppy	Rape	Elevator	Sckroaped	Spunds	Frert
Desire	Slaughter	Radiator	Ghoaned	Strumf	Sooge
Loved	Terrible	Taxi	Clid	Meuth	Norl
Sunset	Hostage	Skeptical	Swees	Wrurn	Plished
Kindness	Sickness	Errand	Ghekes	Frisp	Skriend
Intimate	Hate	Ankle	Ghanse	Shreils	Blirth
Champion	Suicide	Cord	Whint	Glunge	Frenths
Romantic	Corpse	Barrel	Youge	Ghlerve	Scrept
Sunrise	Gloom	Anxious	Sprect	Stive	Scried
Angel	Punishment	Reserved	Yomed	Whurfs	Jict
Happy	Toxic	Truck	Ghroothe	Ghots	Dwoffed
Rescue	Poverty	Bandage	Skoove	Shwoared	Sprait
Victory	Funeral	Glacier	Spronged	Sman	Gwieves
Snuggle	Thief	Thermometer	Sloints	Dasps	Thwebbs
Cash	Nightmare	Avenue	Smurge	Ghafe	Tesk
Birthday	Rejected	Reptile	Glend	Phreched	Shwoast
Improve	Crushed	Basket	Twunks	Phlice	Twoursed
Orgasm	Execution	Scissors	Kurked	Veafs	Whenked
Sweetheart	Frustrated	Foot	Spilge	Skroaked	Prirge
Savior	Murderer	Startled	Sproised	Frald	Blinned
Pleasure	Cancer	Locker	Stec	Trels	Twuin
Sexy	Stupid	Corridor	Skerth	Thrig	Brelm
Joy	Hatred	Seat	Veeced	Skousts	Sckeethed
Baby	Rotten	Pamphlet	Greems	Kreets	Trurn
Waterfall	Jail	Patent	Wralve	Grynx	Zoy
Reward	Death	Contents	Crigs	Gwope	Slurt
Secure	Tumor	Chair	Zinned	Shringed	Druds
Graduate	Burial	Column	Rharm	Flurs	Drymed
Paradise	Depression	Serious	Clauced	Slea	Krorce
Valentine	Unhappy	Indifferent	Splocs	Phroud	Jurped
Caress	Traitor	Knot	Caids	Gwirst	Dwoped
Diploma	Ache	Spray	Phruths	Glurf	Skusp
Pillow	Selfish	Month	Chigs	Shrolfed	Shorth
Treasure	Ulcer	Headlight	Flods	Ghourned	Skreigns
Carefree	Hurt	Passage	Drarve	Sckraise	Prike
Leader	Loneliness	Square	Sloined	Mawped	Trawped
Admired	Pain	Trunk	Gweeled	Gwells	Twames
Fun	Lonely	Bowl	Trut	Knords	Jorks
Triumph	Alone	Metal	Drength	Knaved	Snoule
Spouse	Killer	Building	Thist	Raluse	Hoost
Affection	Vomit	Paper	Mege	Knurk	Spridged
Glory	Infection	Mantel	Struff	Plalc	Ghinched
Adorable	Violent	Tower	Splumed	Daif	Pliped
Joyful	Debt	Concentrate	Twisc	Yolf	Throoves
Comedy	Distressed	Non-chalant	Sckrets	Thwolfed	Craides
Handsome	Poison	Coarse	Rhuiche	Wask	Snilmed
Rainbow	Divorce	Elbow	Skrebe	Peagued	Plued
Fame	Cruel	Cork	Scotch	Trarve	Yinge
Bright	Grief	Stool	Stalmed	Doak	Thrunks
Liberty	Helpless	Engine	Swarched	Ghalp	Splooze
Luscious	Disloyal	News	Daths	Wulps	Thrynes
Delight	Illness	Butter	Fuds	Swoin	Blauze
Wise	Disgusted	Statue	Skeaps	Glirth	Sunged
Flirt	Drown	Hats	Thoathe	Ghunds	Screts
Satisfied	Useless	Wagon	Joots	Sparb	Stawse
Beach	Demon	Obey	Stronze	Shwunks	Scracked
Triumphant	Troubled	Activate	Trets	Twofts	Koove
Holiday	Despairing	Glass	Ning	Gwilched	Twounged
Aroused	Agony	Machine	Dwelse	Ghwoan	Twaifs
Proud	Misery	Lamp	Chonged	Troz	Glayed
Talent	Mutilate	Finger	Kouths	Shriefed	Cooths
Adventure	Hardship	Tool	Roist	Stoft	Bloam
Honor	Defeated	Poster	Shrumps	Ghlawked	Yeight
Beauty	Victim	Eggs	Yitched	Smilts	Smouthed
Friendly	Paralysis	Kettle	Cenge	Cleeled	Sckroche
Honest	Accident	Material	Phroch	Screlds	Dopped
Thoughtful	Stress	Hammer	Preined	Smorched	Cloils
Acceptance	Insult	Fork	Kurned	Glormed	Twirds
Wedding	Mad	Phase	Phusk	Stirds	Knerm
Terrific	Sick	Iron	Wripts	Rholk	Spreiled
Engaged	Insecure	Violin	Kreeled	Ghwurfs	Skuns
Gold	Sad	Lawn	Prines	Spisp	Knaphed
Diamond	Ambulance	Arms	Chites	Jeaked	Ghren
Merry	Afraid	Appliance	Vinsed	Twepe	Grirm
Applause	Rage	Curtains	Horged	Phrerge	Vorled
Success	Terrified	Hydrant	Strimn	Alks	Ghrodes
Sunlight	Starving	Icebox	Shrogues	Pempt	Cloached
Trophy	Bankrupt	Patient	Spokeed	Skrouds	Sckrersed
Cute	Betray	Non-sense	Sckrouled	Parps	Scrupped
Christmas	Assault	Theory	Thweath	Croids	Henched
Gift	Anguished	Pencil	Thwulged	Phriege	Smests
Joke	Disaster	Hairdryer	Stards	Gwurls	Crerp
Humor	Headache	Hairpin	Clouch	Kevved	Frong
Confident	Tragedy	Fabric	Strebbs	Gwexts	Gwatch
Enjoyment	Dreadful	Inhabitant	Funge	Sinsed	Choys
Luxury	Depressed	Umbrella	Smits	Ghifs	Lisk
Cuddle	Filth	Utensil	Volf	Smaught	Skolve
Kiss	Bomb	Stove	Gleps	Sckrymn	Flemmed
Cheer	Torture	Aloof	Feaks	Spost	Wrurke
Loyal	Anger	Journal	Shrife	Treen	Wrard
Lucky	Crash	Rattle	Wruints	Skruilds	Stuild
Thrill	Roach	Reverent	Flols	Spodes	Stuids
Laughter	Maggot	Slush	Whurch	Sckrairs	Trenced
Excitement	Upset	Quart	Shoists	Zauled	Fleash
Profit	Burdened	Subdued	Spleeve	Neaped	Bluits
Promotion	Lice	Chin	Crights	Krunds	Skealed
Ecstasy	Failure	Blase	Thurves	Shreft	Shalved
Hug	Injury	Kerchief	Phull	Phlurs	Tamming
Riches	Despise	Vest	Shraim	Sploids	Chanx

Complete list of positive words, negative words, neutral words, and non-words used as prime targets in the lexical decision component of ALPS run following the 2008 US presidential election.

Stimuli in all experiments were presented on an LCD monitor (1024 × 768 resolution) approximately 60 cm from the participant. This resulted in face primes that subtended a visual angle of approximately 6–7° (horizontal) and 7–8° (vertical).

### Procedure

Participants completed ALPS as a measure of implicit racial bias (Figure 2). Similar in nature to the IAT (Greenwald et al., 1998), and the Bonafide Pipeline (Fazio et al., 1995), ALPS (Lebrecht et al., 2009) was designed to probe attitudes that are automatic and exempt from conscious control. We predict that affective priming occurs in ALPS as the result of participants automatically assigning each face prime with a positive or negative valence, which can in turn facilitate or inhibit response speed on a subsequent lexical decision containing positive and negative words. The perceptual component to ALPS—processing a visually-presented face—allows us a methodological tool to link face perception with affective and social cognition (Lebrecht et al., 2009).

A trial begins by briefly presenting participants with a face for 250 ms. In the present student the critical variable was whether this face prime is Black or White. After a 200 ms inter-stimulus interval (ISI), participants are presented with a target letter-string that is either positive (e.g., “love”), negative (e.g., “hate”), neutral (e.g., “tree”), or a non-sense word (e.g., “malk”). The participant is required to make a binary word/non-sense word decision on the target letter-string by making a keyboard response. This letter-string remains on the screen until the participant has made their response or for 1000 ms, whichever is shorter, at which point a fixation cross is displayed during a 1000 ms inter-trial interval (ITI) prior to the start of the next trial.

## Results

Analyses only include response times that fell within two standard deviations of the mean for each individual participant—a standard method used in the behavioral sciences to correct for skewed response time distributions or outliers. We empirically examined whether this transformation did what we intended—take skewed RT data and render it as a more normal distribution. To that end, we computed a skewness measure for each participants' RTs before and after removing responses beyond ±2 standard deviations. While the raw data showed a mean skewness of 2.94; the transformed data showed a mean skewness of 1.22. Thus, the data subject to further analysis was much closer to normal as preferred for parametric statistical analyses, although this transformation does not necessarily reduce correlational biases in our response time data (Sriram et al., 2010). One alternative is to take the log of individual response time prior to computing means; however, when we transformed the data in this manner we observed that although “long-tail” distributions in our data became less skewed, other distributions, for example those that had long tails in both directions, were not similarly corrected and, overall, non-normalities in the data were not uniformly reduced.

Statistical tests were performed on the dependent measure of group averages of facilitation scores, which were normalized response times determined as follows: response times within a given condition were computed for each individual participant by subtracting their mean response times for positive or negative words from their mean response time for neutral words. For example, the degree of facilitation in response time for black face primes and positive words was calculated by: [black face primes/neutral word trials—black face primes/positive word trials]. Similar calculations were done for negative word trials and for white face primes. All analyses were based on the following independent factors: Pre-/post-election for time of testing (between subjects); US/Canada participant groups (between subjects); Race of Prime for face images (black/white; within subjects); Valence of Word for lexical decision (positive/negative; within subjects).

**Figure 4:**
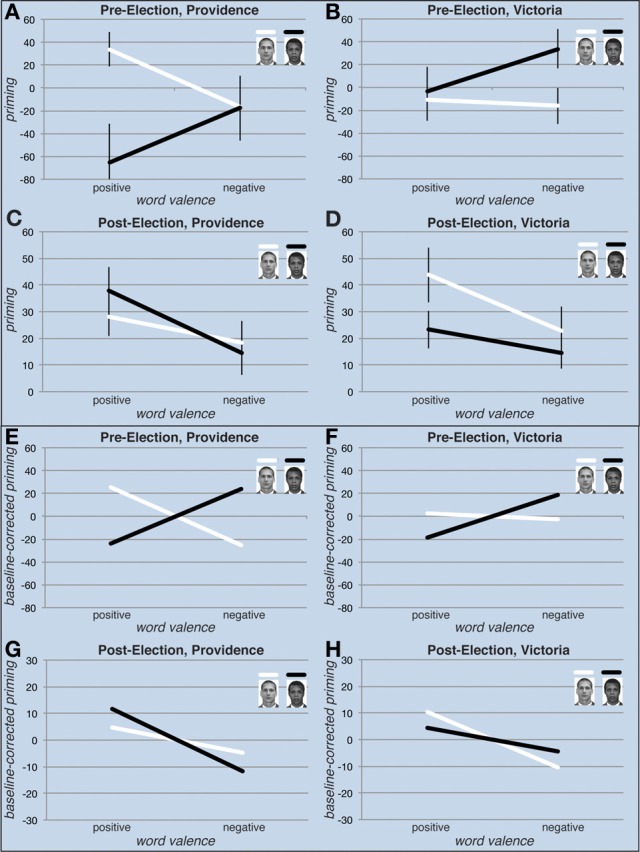
**Pre-election vs. post-election priming results**. To more clearly illustrate the interactions of interest, raw priming effects with between-participant standard errors are shown in **(A–D)**, while baseline-corrected priming effects—in which the mean for each race face prime condition is subtracted from the raw priming data—are shown in **(E–H)**. Baseline-corrected data is shown without the between-participant standard errors in that the critical illustrative point for these panels is the within-participant interactions. Before the 2008 US presidential election participants in both the US and Canada showed a crossover interaction in which black and white faces differentially primed positive and negative words **(A,B,E,F)**. Consistent with the extant literature on implicit attitudes, white faces primed positive words more than negative words, while black faces primed positive words less than negative words. After the 2008 US presidential election participants in the US showed a reversal in their crossover interaction, with black faces also priming positive words more than negative words **(C,G)**. In contrast, participants in Canada showed a crossover interaction consistent with their pre-election results, albeit one in which, although not significant, black faces primed positive words slightly more than negative words **(D,H)**.

Pre-election both US and Canadian participants exhibited the expected pattern of responses consistent with the majority of implicit racial attitudes studies: associating black faces with a negative valence and white faces with a positive valence (Figure 4). The 2-way interaction for Race of Prime × Valence of Word was [*F*_(1, 33)_ = 4.53, *p* < 0.05, η^2^_*p*_ = 0.12] for US participants and [*F*_(1, 23)_ = 3.63, *p* = 0.069, η^2^_*p*_ = 0.14] for Canadian participants—these interaction effects are illustrated (**E**) and (**F**) of Figure 4. Note that we observed no significant interaction in the response times between the two participant populations pre-election—the 3-way interaction for Race of Prime × Valence of Word × US/Canada was [*F*_(1, 56)_ = 909, *p* = 0.34] and the Race of Prime × Valence of Word interaction across US and Canadian participants was [*F*_(1, 56)_ = 5.87, *p* < 0.05, η^2^_*p*_ = 0.10]. These interactions reflected the same pattern typically found in other studies of implicit attitudes: white faces primed positive words significantly more than black faces primed positive words across US and Canadian participants [*t*_(57)_ = 2.02, *p* < 0.05, *d* = 0.41]. More specifically, for US participants, pre-election, black faces primed negative words more than positive words, although this difference was not significant, [*t*_(33)_ = −1.14, *p* = 0.26, *d* = −0.26], while for Canadian participants, pre-election, black faces primed negative words more than positive words [*t*_(23)_ = −2.29, *p* < 0.05, *d* = −0.39]; for US participants, pre-election, white faces primed positive words more than negative words [*t*_(33)_ = 2.15, *p* < 0.05, *d* = 0.45], while for Canadian participants, pre-election, white faces did not prime positive words more than negative words [*t*_(23)_ = 0.35, *p* = 0.73, *d* = 0.06].

Given that we did not expect to find differences between data collected pre- and post-election, we continued to refine ALPS and, post-election, employed a new, larger word set that was intended to increase the sensitivity of ALPS to implicit attitudes (Bradley and Lang, 1999). As discussed in elsewhere in this paper, these refinements led to some confounds in how strongly we can interpret our unexpected findings—ideally, identical (and refined) image and word sets would have been used pre- and post-election in both the US and Canada. However, even in the context of these less-than-desirable variations among stimuli we are hard pressed to identify mechanistic reasons as to why these stimulus differences would have resulted in the shifts we observed in implicit attitudes. For example, the black faces used post-election are more negative in appearance as compared to the black faces used pre-election in Providence, yet it was post-election that black faces were associated with more positive words. One difference between word sets that we should highlight is that the new word set resulted much faster responses to positive words regardless of prime type. This is reflected in a significant main effect of word valence in the combined post-election, US and Canadian dataset [*F*_(1, 63)_ = 21.5, *p* < 0.001, η^2^_*p*_ = 0.25]. That is, in the post-election experiments, regardless of the prime, participants were faster overall to identify a word as a word if it was positive as compared to if it was negative. Although this main effect does not impact the significance of our overall findings, it does make them harder to interpret. This is reflected in the different scales used for plotting priming scores in the pre- and post-election graphs (Figure 4). At the same time, the critical comparison between our post-election US and post-election Canadian participants is based on data collected using the same word set, and therefore measured on the same scale.

The central question is whether US and Canadian implicit attitudes toward black faces changed over time. As already stated, before the 2008 US presidential election, participants in the US and in Canada both showed a negative association with black faces. However, after the election, US participants showed a *reversal* in the attitudes they associated with black faces (Figures 4C,G). The 2-way interaction for Race of Prime × Valence of Word was [*F*_(1, 28)_ = 1.79, *p* = 0.19] for US participants and [*F*_(1, 35)_ = 2.60, *p* = 0.12] for Canadian participants—these interaction effects are illustrated (**G**) and (**H**) of Figure 4. Critically, in contrast to our pre-election results, we observed a significant interaction in the response times between the two participant populations post-election—the 3-way interaction for Race of Prime × Valence of Word × US/Canada was [*F*_(1, 63)_ = 4.31, *p* < 0.05, η^2^_*p*_ = 0.06]. More specifically, this interaction reflected a pattern that is not typically found in studies of implicit attitudes: for US participants, post-election, black faces primed positive words more than negative words [*t*_(28)_ = 3.57, *p* < 0.001, *d* = 0.52], while for Canadian participants, post-election, black faces did not prime positive words more than negative words [*t*_(35)_ = 1.47, *p* = 0.15, *d* = 0.29]; for US participants, post-election, white faces did not prime positive words more than negative words [*t*_(28)_ = 1.36, *p* = 0.18, *d* = 0.24], while for Canadian participants, post-election, white faces primed positive words more than negative words [*t*_(35)_ = 3.29, *p* < 0.01, *d* = 0.36]. The fact that we did not find a significant difference for US participants in the degree to which white faces primed positive words as compared to negative words is somewhat puzzling (although the trend is still in the expected direction)—we speculate that this may have been an artifact of much faster responses associated with positive words in the post-election word set. As addressed in Lebrecht (2012) shorter response times in the lexical decision component of ALPS lead to reduced priming effects.

This reversal in implicit attitudes with respect to black faces in US participants was reflected in a significant 3-way interaction for Race of Prime × Valence of Word × Pre-/Post-Election [*F*_(1, 61)_ = 4.84, *p* < 0.05, η^2^_*p*_ = 0.07] (Figures 4E,G). In contrast, Canadian participants did not show a significant 3-way interaction for Race of Prime × Valence of Word × Pre-/Post-Election [*F*_(1, 58)_ = 2.24, *p* = 0.14] (Figures 4F,H). That is, for Canadian participants, the pattern in measured attitudes did not change significantly before and after the 2008 election. However the 4-way interaction for Race of Prime × Valence of Word × US/Canada × Pre-/Post-Election was not significant [*F*_(1, 119)_ = 2.10, *p* = 0.15]—although since the Canadian group was trending in the same direction as the US group post-election, one might not expect this interaction to be significant. Indeed, for Canadian participants the shift in a positive direction with respect to the degree to which black faces primed positive words hints that whatever societal factors influenced our US participants' implicit attitudes, the same factors may have been, to a lesser extent, in play for our Canadian participants. Supporting this conjecture, although entirely anecdotal, many Canadians report that there was extremely strong support for President Obama in the run up to the 2008 election. At the same time, we note that a record setting $745 million was spent by the Obama campaign in the 2008 election (Queen and Hilland, 2009)—money spent specifically to influence US voters' beliefs about the candidates. However, we should be very cautious in this interpretation in that both the US and the Canadian participants were assessed using new sets of face stimuli and words—thus, any common shift may have been partially attributable to these factors.

Reviewing our results, our strongest evidence for a shift in implicit attitudes in US participants, but not Canadian participants, across the 2008 US presidential election is a significant 3-way interaction for Race of Prime × Valence of Word × US/Canada following the election. In contrast, this same 3-way interaction was not significant before the election. Of particular note, during the post-election time period in which these data were collected in the US, President Obama's US approval ratings ranged from 62–66% (Woolley and Peters, 2013); similarly, during the time period in which these data were collected in Canada, President Obama's US approval ratings^3^ ranged from 57–61% (Woolley and Peters, 2013). Both of these ranges are quite high relative to the mid-to-low 40's approval ratings typically observed throughout 2010 until the Fall of 2012—leading up to and following the 2012 election, his approval ratings rose slightly into the low 50's (Woolley and Peters, 2013). As such it would be difficult to recreate the positive light in which President Obama was regarded following the 2008 election.

## Discussion

Across four laboratory assessments of implicit racial attitudes, between 2007 and 2009, we observed a reversal in the implicit attitudes associated with black faces for US participants *only*; surprisingly, Canadian participants did not show a similar change in attitudes. Notably, our first two experiments were run prior to November of 2008 and our second two experiments were run after November of 2008. Thus, later in time, US participants implicitly associated black faces with positively valenced words, whereas earlier they had implicitly associated black faces with negatively valenced words. In contrast, over the same time period Canadian participants continued to implicitly associate black faces with negatively valenced words. In and of itself, this pattern is rather puzzling. However, as with the general population, we were personally subject to the high saliency of President Obama's election in November of 2008. In this context, it seemed natural for us to speculate that this historic event had some impact on the measured implicit racial attitudes of our participants.

Reinforcing our conjectures at the time, a special section assessing the impact of President Obama's election appeared in the *Journal of Experimental Social Psychology* (Cooper, 2009). Perhaps most striking among the several articles was Plant et al.'s report that they had observed a post-election decrease in implicit racial attitudes toward African-Americans as measured by the IAT (Plant et al., 2009). Similarly, another study reported that the election of President Obama influenced test-taking performance of African-American and Caucasian students by chronicling performance of African-American students before and after the election (Marx et al., 2009). Even further afield, but perhaps explainable through a change in negative implicit attitudes toward African-Americans, some criminologists and economists noted a possible “Obama Effect” in a decrease in violent crime in the US in 2009 and 2010 (Dubner, 2011). Criminologist Alfred Blumstein commented that “The one striking event that comes to mind is the inauguration of our first African-American president, a particularly salient event in this context … ” and cites data that indicate a higher reduction in the number of arrests of African-Americans as compared to Caucasians—indeed, for drug use offenses, the reduction was 3.3% for African-Americans, while there was actually an increase of 2.0% over the same period for Caucasians (Dubner, 2011).

Thus, we felt and continue to feel that is reasonable to present our findings in the same context as other social cognition studies addressing similar questions. At the same time, we acknowledge the limitations of any hypothesis positing a specific real-world event as a causal mechanism must necessarily remain speculative, in that it is impossible to recreate/replay transient, historical events or precisely mimic their structure in a laboratory setting. That is, there is no way we can draw a causal link between our pre- and post-election measurements of implicit attitudes and any specific intervening event, highly-salient and theory-relevant or not. This concern becomes even more real when we consider the recent results presented in Schmidt and Nosek (2010). As noted earlier, these authors report that a large-*N* analysis of implicit racial attitudes as measured by the IAT revealed no shift in attitudes across the time period before and after the 2008 US presidential election. More fine-grained analyses also support this general finding: for example, the level of accessibility to Barack Obama, as measured by the number of daily news articles containing the word “Obama,” did not reliably influence daily measures of implicit racial attitudes. Similarly, analyses predicated on social group membership or political orientation revealed only minimal shifts in implicit racial attitudes, as did analyses centering around important dates in President Obama's candidacy and election (Schmidt and Nosek, 2010). With respect to Plant et al.'s (2009) finding of decreased levels of implicit racial bias toward African-Americans, Schmidt and Nosek (2010) found no evidence that targeted participant samples meant to mirror those used in Plant et al. (2009) exhibited a similar decrease in levels of implicit bias. To address this discrepancy, Schmidt and Nosek offer two explanations. First, it is possible that the decrease observed in Plant et al. (2009) is a Type I error (given the large preponderance of evidence Schmidt and Nosek argue that the appropriate null assumption is that participants will show positive bias toward Caucasians and negative bias toward African-Americans). Second, it is possible that unknown “situational” factors may have come into play in the laboratory collection of data in Plant et al. (2009; e.g., posters or other media in the laboratory that conveyed counter-stereotypical exemplars or ideals). Acknowledging that these two alternative explanations are reasonable in and of themselves, we note that our results are consistent with Plant et al.'s original explanation for their observed decrease in levels of implicit bias. However, in contrast to our findings, Plant et al. did not report, as we do, a significant *reversal* with respect to implicit attitudes toward African-Americans. Thus, it is possible that Schmidt and Nosek's alternative accounts of Plant et al.'s result remain valid, but do not necessarily apply to the findings we report here.

What factors then, might account for our results *vis a vis* those reported in Plant et al. (2009) and Schmidt and Nosek (2010)? Although like Plant et al. (2009) we collected our data in a laboratory setting, we are skeptical that this factor alone is sufficient to explain either a decrease or a reversal in implicit attitudes. In particular, we are confident that our laboratory contexts neither implicitly or explicitly conveyed “egalitarian ideals” (Schmidt and Nosek, 2010). Thus, we concur with Schmidt and Nosek's point that “there is no reason to expect that the effect of Obama's candidacy could only be observed when participants visited a laboratory.” In this light, we view the most plausible explanation for our results as a concatenation of locale and measurement instrument. With regard to the former, we have already noted that the Providence and Brown University populations were highly supportive of President Obama and that some of the iconic imagery of his campaign originated there. With regard to the latter, it is self-evident that the IAT and ALPS are different tools for assessing implicit attitudes. As such, it seems likely that they tap into different cognitive mechanisms and may produce discrepant results depending on a wide variety of factors. More specifically, the IAT relies on a form of response competition between two active concepts that is likely related to the classic “Stroop” paradigm (Klauer, 1997), whereas ALPS relies on fluency within the lexicon that is likely related to semantic (Meyer and Schvaneveldt, 1971) or affective priming (Klauer, 1997). Indeed, there is evidence that affective lexical priming instruments such as ALPS rely on a prime-target relationship whereby automatic processing of the prime “spreads” to other lexical entries that share the same valence direction (Klauer, 1997; Wentura, 2000). In contrast, instruments such as the IAT may rely on the independent evaluation of the two competing potential responses and, depending on the task, valence congruency may either facilitate or interfere with the participant's actual evaluative responses (Klauer, 1997). Although clearly a good deal more work is required to pinpoint differences in the cognitive mechanisms underlying the IAT and ALPS, we speculate that because there is no overt reference to social group or affect within ALPS, any effects of valence are likely to be entirely unconscious—as such ALPS may be somewhat more sensitive to subtleties in some aspects of implicit attitudes. Finally, as we have already noted, there is a striking congruency between the media images used in the presidential campaign (Figure 1) and the structure of ALPS (Figure 2)—it is possible that we unintentionally stumbled onto the ideal instrument for assessment the effect of this particular media.

We should note—as already discussed—that there are two potential confounds that cloud any interpretation of our data: the change in word set and the change in face set pre- and post-election. Although the new word set appears to have produced much faster responses to positive words as compared to negative words, it is difficult to envision how this effect might have interacted with the race of the faces used as primes or with any particular participant group. With respect to the new face set, two logical points argue against any idiosyncratic influence for a particular set of faces for any particular participant group. First, *different* faces were used for US and Canadian participants pre-election—yet these two pre-election participant groups showed the *same* pattern of results. In contrast, the *same* faces were used for US and Canadian participants post-election—yet these two post-election participant groups showed *different* patterns of results. Second, the Department of Corrections face stimuli used for both post-election participant groups were, if anything, more negative in overall appearance relative to more “typical” experimental face stimuli (e.g., the faces used for the US pre-election participant group were Brown University community members—mostly undergraduate students). As such, one might have expected stronger negative implicit attitudes for such faces—yet the only observed reversal of the typically-seen negative implicit attitudes for black faces occurred with these nominally *more* negative faces. Thus, there appears to be little evidence to support an argument that differences across the face stimuli used in each condition underlie any of our theoretically-relevant effects.

Finally, we should note that our sample sizes were relatively small as compared to those reported in Schmidt and Nosek (2010), but only slightly smaller than those reported in Plant et al. (2009). However, the sample size of our study is not particularly atypical or small as compared to the extensive extant literature in cognitive psychology (or even social cognition until recently). Indeed, many priming studies of non-social processes, for example, lexical priming, have used equivalent or fewer subjects per a condition (e.g., Meyer and Schvaneveldt, 1971, used 12 participants per an experiment). Thus, *de facto*, there is no reason to assume that our sample size is small in and of itself. That being said, we want to be up front about the relatively low power of our study, where power is a result of not just sample size, but the strength of the effect(s): to be clear, the effects we report are relatively fragile and we agree that additional samples would be desirable. However, as discussed in detail, in our rather unusual case, this is not possible.

Beyond these non-theory-relevant factors—context, different measurement instruments, different stimulus sets, size of sample—perhaps the most compelling aspect of our study is that we were able to compare two populations that were, nominally, differentially exposed to media about President Obama both pre- and post-election. Thus, we were able to establish that the changes in implicit attitudes observed in our US participants were not found in a non-US population with arguably less exposure to US election coverage. As mentioned earlier, several studies have shown that exposure to salient counter-examples can serve as a significant moderator of racial bias in short-term training and in group-level gender bias differences (Kawakami et al., 2000; Blair, 2002; Dasgupta and Asgari, 2004). Insofar as our assessment of post-election behavior included *no* experimenter-directed instruction or experimental construct—either implicit or explicit—that would indicate that either participant population should suppress implicit negative racial stereotypes, we posit that our results speak to the influence of repeated exposure to a salient counter-stereotypic real-world exemplar. Put another way, in order for implicit attitudes to change measurably at the group level (i.e., for a significant sample of the tested Brown undergraduates), some salient event must have precipitated a considerable change in the overall stereotypes associated with the racial group in question. Of course, we have no way of directly measuring what might have caused this real-life change across our groups of participants. However, as with others in the field, we can retrospectively theorize as to what event in the past several years could be so significant, and unique to our US participants, as to produce a reversal in a student population's implicit attitudes toward African-Americans. It is our conjecture that the election of the United States' first African-American president concomitant with extensive media coverage was the primary factor.

In order to better understand how one exemplar, Barack Obama, might have facilitated the reversal of implicit attitudes attributed to an entire race, we focus on a specific perceptual mechanism: the “Other Race Effect” (ORE) (Malpass and Kravitz, 1969). The ORE is defined as an individual's superior ability to *visually* individuate faces drawn from the race which that individual was most frequently exposed to during development, as compared to faces drawn from a race they encountered less frequently (Bar-Haim et al., 2006). Most commonly, this effect is experienced as other-race faces looking more similar to one another (despite no difference in actual image similarity relative to the familiar-race faces). Along with strong evidence to suggest that the ORE is a perceptual effect (Tanaka et al., 2004; Tanaka and Pierce, 2009; McGugin et al., 2011), there is evidence to suggest that it also impacts socio-cognitive mechanisms, such as implicit racial attitudes (Ferguson et al., 2001; Hugenberg et al., 2007; Lebrecht et al., 2009). We suggest that the ORE and implicit racial attitudes are linked in that if observers are poorer at perceptually differentiating other-race faces, they are less likely to make individual-level social attributions and more likely to make group-level social attributions. This decreased likelihood of observers making individual social attributions with black faces presents a challenge to breaking down group-level attributions. Stereotypes typically generalize across much of a group, and perceptual processing of other-race faces may in fact reinforce these broad generalizations. That is, if other-race faces are perceptually more similar to one another, it is more likely that a single label will be applied across individuals of that race.

**Figure 5:**
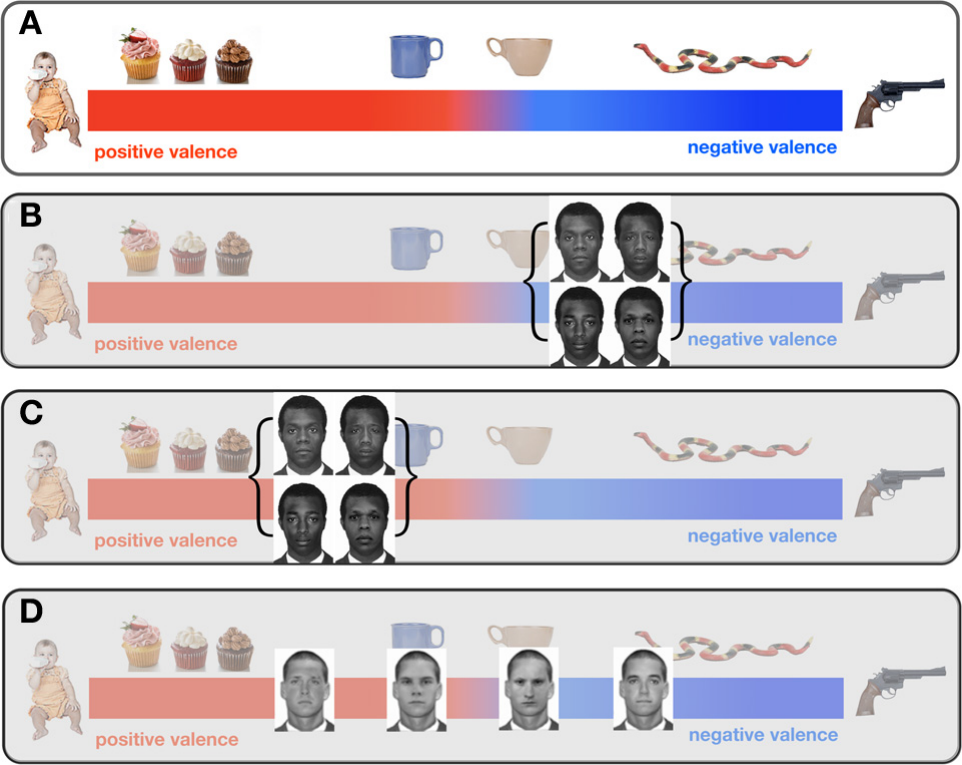
**The valence Continuum**. The valence continuum **(A)** illustrates the hypothesis that faces and objects are automatically evaluated with respect to a metric of positive or negative based on affective associations. Valences for faces and objects can be organized on a continuum that ranges from strongly positive to strongly negative. Pre-election, on average, black faces elicited negative associations—supported by black faces priming negative words more so than positive words. As illustrated in **(B)**, this posits that, pre-election, black faces were clustered at the negative end of the valence continuum. Note that this clustering is, at least in part, a product of the ORE, namely that for the majority of Caucasian participants black faces look more similar to one another as compared to white faces. Thus, the affective associations and concomitant implicit biases overlap for Caucasians encountering African-Americans. **(C)** Notably for US participants only the position of this cluster shifts to the positive end of the continuum post-election, as suggested the data reported in this paper. **(D)** Similar clustering would not seen for Caucasian participants looking at white face primes in that participants are better able to perceptually discriminate between own-race faces, and so associate individual primes with lower overlapping valences. This greater variability in valence for own-race individuals may help account for why we observed a more consistent positive association with black faces as compared to white faces for participants in Providence.

We further hypothesize that implicit attitudes arise when affective associations are reactivated during face perception: faces are automatically evaluated as positive or negative. Because other-race faces are perceptually more similar, the affective associations that are reactivated in perception are more poorly differentiated and attitudes generated from these associations will be more likely to overlap. This proposed framework is supported by Verosky and Todorov's (2010) finding that participants rate novel faces (20–35% morphs) more positively or negatively based on their perceptual similarity to learned affective faces. In the context of a valence continuum, other-race faces will then cluster within a narrower range relative to own-race faces (Figure 5). Pre-election, we posit that black faces were localized toward the negative end of the continuum due to negative racial stereotypes (Fazio et al., 1995; Greenwald et al., 1998) and the predominantly negative news coverage of African-American individuals (Entman, 1994). Post-election for our US participants, this cluster may be anchored around a more positive location along the continuum, as black faces may have been perceived as similar to President Obama's highly positive face (Figure 5). We posit that observers typically show implicit *positive* attitudes for own-race faces based on the same principles. That is, in contrast to the perceptual clustering of other-race faces, most observers should have sufficient perceptual expertise to accurately and automatically differentiate own-race faces (Lebrecht et al., 2009). As our participants were Caucasian, we posit that they perceived own-race faces (i.e., Caucasian faces) to be more spread out along the valence continuum (Figure 5). Yet oddly, in our study we do not consistently observe the expected positive attitudes for white faces. Our admittedly *post-hoc* explanation for this finding may be that the faces used as stimuli (both black and white) were particularly negative in appearance given that they were taken from a Department of Corrections face database (of note, this bias would work against our actual observation of a shift toward positive attitudes for black faces drawn from the same face database).

Finally, we should also acknowledge that a limitation of our present study (as well as in Plant et al., 2009) is that we have no means for assessing media exposure (positive or negative) as experienced by students at Brown University or at the University of Victoria in Canada. We speculate that while Canadian news organizations certainly did cover the US election, there were a variety of factors that may have contributed to change we observed: extremely positive coverage of President Obama's election campaign at Brown, overwhelmingly strong support for President Obama on campus, the fact that President Obama's brother-in-law was the head coach of the Brown basketball team from 2006–2008 (he resigned from this position to help with the campaign), an African-American president at Brown since 2001, and, finally, Providence as the home of the creator of the striking Barack Obama “HOPE” images (Figure 1). Thus, Brown—a notably liberal campus with direct ties to President Obama—may have been the ideal “petri dish” for observing any shift in the implicit attitudes arising from the 2008 election. Of course, although this reported shift in implicit racial attitudes appears to be a positive change, we wish to reiterate that a single label, positive or negative, is clearly inadequate to characterize individuals within any race. In that our present results suggest that such labeling is potentially malleable even within non-laboratory contexts, we should be aware that future salient events may influence these and other attitudes as expressed by real-world populations. In this context we hope that as a field we can be opportunistic—assessing, when possible, whether the effects we present here are replicable using ALPS or other measures of implicit attitudes, as well as whether any observed effects are population general or interact with participants' perceptual experiences (e.g., Lebrecht et al., 2009) and/or personal biases.

### Conflict of interest statement

The authors declare that the research was conducted in the absence of any commercial or financial relationships that could be construed as a potential conflict of interest.

## Footnotes

^1^In our paper we use of the term “media” to refer both to media coverage, that is, newspapers, TV news, etc., and to paid advertising, that is, TV commercials, print ads, direct mail flyers, etc.

^2^To better control the composition of our participant population with respect to the perception of other-race faces Asian participants were excluded from this study.

^3^We were unable to find a source for US Presidential approval ratings for Canadian residents.

## References

[B1] Bar M. Kassam K. S. Ghuman A. S. Boshyan J. Schmid A. M. Schmidt A. M. (2006). Top-down facilitation of visual recognition. Proc. Natl. Acad. Sci. U.S.A. 103, 449–454 10.1073/pnas.0507062103 16407167 PMC1326160

[B2] Bar-Haim Y. Ziv T. Lamy D. Hodes R. M. (2006). Nature and nurture in own-race face processing. Psychol. Sci. 17, 159–163 10.1111/j.1467-9280.2006.01679.x 16466424

[B3] Barrett L. F. Bar M. (2009). See it with feeling: affective predictions during object perception. Philos. Trans. R. Soc. Lond. B Biol. Sci. 364, 1325–1334 10.1098/rstb.2008.0312 19528014 PMC2666711

[B4] Blair I. V. (2002). The malleability of automatic stereotypes and prejudice. Pers. Soc. Psychol. Rev. 6, 242 10.1207/S15327957PSPR0603_8

[B5] Bradley M. M. Lang P. J. (1999). Affective Norms for English Words (ANEW): Stimuli, Instruction Manual, and Affective Ratings (Technical Report C-1). Gainesville, FL: The Center for Research in Psychophysiology

[B6] Cave D. (2008, November 7). Generation O gets its hopes up. The New York Times. Retrieved from: http://www.nytimes.com/2008/11/09/fashion/09boomers.html

[B7] Cooper J. (2009). A special flashreports section on the election of Barack Obama. J. Exp. Soc. Psychol. 45, 952–969 10.1016/j.jesp.2009.05.007

[B8] Correll J. Park B. Judd C. M. Wittenbrink B. Sadler M. S. Keesee T. (2007). Across the thin blue line: police officers and racial bias in the decision to shoot. J. Pers. Soc. Psychol. 92, 1006–1023 10.1037/0022-3514.92.6.1006 17547485

[B9] Dasgupta N. Asgari S. (2004). Seeing is believing: exposure to counterstereotypic women leaders and its effect on the malleability of automatic gender stereotyping. J. Exp. Soc. Psychol. 40, 642–658 10.1016/j.jesp.2004.02.003

[B10] Dasgupta N. Greenwald A. G. (2001). On the malleability of automatic attitudes: Combating automatic prejudice with images of admired and disliked individuals. J. Pers. Soc. Psychol. 81, 800–814 10.1037/0022-3514.81.5.800 11708558

[B11] Dubner S. J. (2011). Freakonomics quorum: why, during a bad economy, does crime continue to fall? Freakonomics Blog [Web page]. Retrieved from: http://www.freakonomics.com/2011/06/08/freakonomics-quorum-why-during-a-bad-economy-does-crime-continue-to-fall/

[B12] Eberhardt J. L. Goff P. A. Purdie V. J. Davies P. G. (2004). Seeing black: race, crime, and visual processing. J. Pers. Soc. Psychol. 87, 876–893 10.1037/0022-3514.87.6.876 15598112

[B13] Entman R. M. (1994). Representation and reality in the portrayal of blacks on network television news. J. Q. 71, 509 10.1177/107769909407100303

[B14] Fazio R. H. Jackson J. R. Dunton B. C. Williams C. J. (1995). Variability in automatic activation as an unobtrusive measure of racial attitudes: a bona fide pipeline? J. Pers. Soc. Psychol. 69, 1013–1027 10.1037/0022-3514.69.6.1013 8531054

[B15] Fedor L. (2008, November 12). Around the Ivies, celebrations of election. Brown Daily Herald. Retrieved from: http://www.browndailyherald.com/2.12237/around-the-ivies-celebrations-of-election-1.1668794

[B16] Ferguson D. P. Rhodes G. Lee K. Sriram N. (2001). ‘They all look alike to me’: Prejudice and cross-race face recognition. Br. J. Psychol. 92, 567–577 10.1348/000712601162347 11762861

[B17] Green A. R. Carney D. R. Pallin D. J. Ngo L. H. Raymond K. L. Iezzoni L. I. (2007). Implicit bias among physicians and its prediction of thrombolysis decisions for black and white patients. J. Gen. Intern. Med. 22, 1231–1238 10.1007/s11606-007-0258-5 17594129 PMC2219763

[B18] Greenwald A. G. McGhee D. E. Schwartz J. L. (1998). Measuring individual differences in implicit cognition: the implicit association test. J. Pers. Soc. Psychol. 74, 1464–1480 10.1037/0022-3514.74.6.1464 9654756

[B19] Hugenberg K. Miller J. Claypool H. M. (2007). Categorization and individuation in the cross-race recognition deficit: toward a solution to an insidious problem. J. Exp. Soc. Psychol. 43, 334–340 10.1016/j.jesp.2006.02.010

[B20] Kawakami K. Dovidio J. F. Moll J. Hermsen S. Russin A. (2000). Just say no (to stereotyping): effects of training in the negation of stereotypic associations on stereotype activation. J. Pers. Soc. Psychol. 78, 871–888 10.1037/0022-3514.78.5.871 10821195

[B21] Klauer K. C. (1997). Affective priming. Eur. Rev. Soc. Psychol. 8, 67–103 10.1080/14792779643000083

[B22] Lebrecht S. (2012). “Micro-valences”: *Affective Valence in “Neutral” Everyday Objects.* Ph.D. thesis, Department of Cognitive and Linguistic Sciences, Brown University, Providence, RI, USA

[B23] Lebrecht S. Pierce L. J. Tarr M. J. Tanaka J. W. (2009). Perceptual other-race training reduces implicit racial bias. PLoS ONE 4:e4215 10.1371/journal.pone.0004215 19156226 PMC2627769

[B24] Lieberman M. D. Hariri A. Jarcho J. M. Eisenberger N. I. Bookheimer S. Y. (2005). An fMRI investigation of race-related amygdala activity in African-American and Caucasian-American individuals. Nat. Neurosci. 8, 720–722 10.1038/nn1465 15880106

[B25] Liss E. (2008, November 3). Students support Obama, Herald poll shows. The Brown Daily Herald. Retrieved from: http://www.browndailyherald.com/campus-news/students-support-obama-herald-poll-shows-1.1668972

[B26] Malpass R. S. Kravitz J. (1969). Recognition for faces of own and other race. J. Pers. Soc. Psychol. 13, 330–334 10.1037/h0028434 5359231

[B27] Marx D. M. Ko S. J. Friedman R. A. (2009). The “Obama effect”: how a salient role model reduces race-based performance differences. J. Exp. Soc. Psychol. 45, 953–956 10.1016/j.jesp.2009.03.012

[B28] McGugin R. W. Tanaka J. W. Lebrecht S. Tarr M. J. Gauthier I. (2011). Race-specific perceptual discrimination improvement following short individuation training with faces. Cogn. Sci. 35, 330–347 10.1111/j.1551-6709.2010.01148.x 21429002 PMC3066453

[B29] Meyer D. E. Schvaneveldt R. W. (1971). Facilitation in recognizing pairs of words: evidence of a dependence between retrieval operations. J. Exp. Psychol. 90, 227–234 10.1037/h0031564 5134329

[B30] Nosek B. A. Banaji M. R. Greenwald A. G. (2002). Harvesting implicit group attitudes and beliefs from a demonstration web site. Group Dynamics 6, 101–115 10.1037/1089-2699.6.1.101

[B31] Phelps E. A. (2001). Faces and races in the brain. Nat. Neurosci. 4, 775–776 11477418 10.1038/90467

[B32] Phelps E. A. O'Connor K. J. Cunningham W. A. Funayama E. S. Gatenby J. C. Gore J. C. (2000). Performance on indirect measures of race evaluation predicts amygdala activation. J. Cogn. Neurosci. 12, 729–738 10.1162/089892900562552 11054916

[B33] Plant E. A. Devine P. G. Cox W. T. L. Columb C. Miller S. L. Goplen J. (2009). The Obama effect: decreasing implicit prejudice and stereotyping. J. Exp. Soc. Psychol. 45, 961–964 10.1016/j.jesp.2009.04.018

[B34] Queen J. Hilland C. (2009). 2009 Presidential Campaign Financial Activity Summarized: Receipts Nearly Double 2004 [Web page]. Retrieved from: http://www.fec.gov/press/press2009/20090608PresStat.shtml

[B35] Richeson J. A. Baird A. A. Gordon H. L. Heatherton T. F. Wyland C. L. Trawalter S. (2003). An fMRI investigation of the impact of interracial contact on executive function. Nat. Neurosci. 6, 1323–1328 10.1038/nn1156 14625557

[B36] Schmidt K. Nosek B. A. (2010). Implicit (and explicit) racial attitudes barely changed during Barack Obama's presidential campaign and early presidency. J. Exp. Soc. Psychol. 46, 308–314 10.1016/j.jesp.2009.12.003

[B37] Sriram N. Greenwald A. G. Nosek B. A. (2010). Correlational biases in mean response latency differences. Stat. Methodol. 7, 277–291 10.1016/j.stamet.2009.10.004 20526445 PMC2879027

[B38] Tanaka J. W. Pierce L. J. (2009). The neural plasticity of other-race face recognition. Cogn. Affect. Behav. Neurosci. 9, 122–131 10.3758/CABN.9.1.122 19246333

[B39] Tanaka J. W. Kiefer M. Bukach C. M. (2004). A holistic account of the own-race effect in face recognition: evidence from a cross-cultural study. Cognition 93, B1–B9 10.1016/j.cognition.2003.09.011 15110726

[B40] Trawalter S. Todd A. R. Baird A. A. Richeson J. A. (2008). Attending to threat: race-based patterns of selective attention. J. Exp. Soc. Psychol. 44, 1322–1327 10.1016/j.jesp.2008.03.006 19727428 PMC2633407

[B41] Verosky S. C. Todorov A. (2010). Generalization of affective learning about faces to perceptually similar faces. Psychol. Sci. 21, 779–785 10.1177/0956797610371965 20483821

[B42] Wentura D. (2000). Dissociative affective and associative priming effects in the lexical decision task: yes versus no responses to word targets reveal evaluative judgment tendencies. J. Exp. Psychol. Learn. Mem. Cogn. 26, 456–469 10.1037/0278-7393.26.2.456 10764106

[B43] Willenbockel V. Sadr J. Fiset D. Horne G. O. Gosselin F. Tanaka J. W. (2010). Controlling low-level image properties: the SHINE toolbox. Behav. Res. Methods 42, 671–684 10.3758/BRM.42.3.671 20805589

[B44] Wittenbrink B. Judd C. M. Park B. (2001). Spontaneous prejudice in context: variability in automatically activated attitudes. J. Pers. Soc. Psychol. 81, 815–827 10.1037/0022-3514.81.5.815 11708559

[B45] Woolley J. Peters G. (2013). The American Presidency Project [Web page]. Retrieved from: http://www.presidency.ucsb.edu/data/popularity.php

